# Contralateral trans-spinous base inclined approach using AUSS for two-level lumbar lateral recess and foraminal stenosis: a case report

**DOI:** 10.3389/fsurg.2026.1859210

**Published:** 2026-06-11

**Authors:** Baogui Gao, Fulong Wu, Huiyan Wang, Caimei Meng, Zhongqiang Liu, Longfei Mo, Shiheng Lu, Zhaojian Zhang, Shuying Wei, Xuemei Li, Zhonghua Li, Changze Yu, Genping Gao, Caizhi Liao

**Affiliations:** 1 Department of Spine Surgery, Beihai Hospital of Traditional Chinese Medicine Affiliated to Guangxi University of Chinese Medicine, Beihai, China; 2 Department of Surgery, Batang Subdistrict Hospital, Gangnan District, Guigang City, Guangxi Province, China

**Keywords:** anterolisthesis, CTI-AUSS, lumbar spinal stenosis, multilevel spinal stenosis, spinal canal decompression

## Abstract

**Background:**

Lumbar spinal stenosis (LSS) is common in elderly and can significantly impair quality of life. With advances in minimally invasive spine surgery, techniques such as unilateral biportal endoscopy (UBE) and arthroscopic-assisted uni-portal spinal surgery (AUSS) have become mainstream treatments. However, LSS in elderly often involves complex pathology, including spinal instability and multilevel stenosis. Traditional open surgery carries risks of iatrogenic instability, significant blood loss, and suboptimal clinical outcomes.

**Case presentation:**

A 72-year-old man presented with low back pain radiating to the left lower extremity Physical exam revealed mild left thigh muscle atrophy. Positive femoral nerve stretch test, diminished patellar reflex, and extensor hallucis longus (EHL) strength graded 4/5. Imaging showed mild lumbar scoliosis, grade I anterior spondylolisthesis at L3, and MRI findings of left lateral recess stenosis at L3/4 and L4/5, along with disc herniation, ligamentum flavum hypertrophy, and facet arthropathy. A far-lateral disc herniation at L4/5 was compressing the exiting left L4 nerve root. Given the patient's fragility and surgical risks, we used a novel contralateral trans-spinous base inclined (CTI) approach-AUSS for laminectomy and spinal canal decompression. The patient experienced immediate postoperative pain relief and neurological improvement. Postoperative imaging confirmed effective decompression at both levels with no progression of spondylolisthesis. The facet joint average preservation rate was 93.97%.

**Conclusion:**

The CTI-AUSS technique is a safe and effective option for treating multilevel lumbar lateral recess and foraminal stenosis in elderly. It provides excellent clinical and radiographic outcomes while preserving spinal stability, protecting paraspinal muscles and facet joints, and reducing the risk of postoperative instability or spondylolisthesis.

## Introduction

Lumbar spinal stenosis (LSS) affects 20%–25% of the general population, with increasing prevalence in those over 60 years of age (1). Traditional surgical options include open laminectomy with or without fusion (2), Delta large-channel endoscopy, and unilateral biportal endoscopy (UBE) (3). These techniques expand the spinal canal, decompress neural elements, and relieve clinical symptoms. However, Delta large-channel endoscopy involves significant trauma and complications, while UBE is limited by poor maneuverability, restricted visualization, and iatrogenic instability may occur postoperatively (4).

This report reviews existing surgical strategies for spinal stenosis and describes a novel contralateral trans-spinous base inclined (CTI)-AUSS for treating two-level lateral recess and foraminal stenosis, demonstrating a minimally invasive method for multilevel spinal stenosis.

## Case presentation

A 72-year-old man presented with chronic low back pain (VAS 7) and a two-year history of neurogenic claudication (< 100 meters) with left lower extremity radicular pain (VAS 6). His preoperative JOA score was 28, and ODI was 41. Physical examination revealed mild left thigh atrophy, positive left femoral nerve stretch test, diminished left patellar reflex, and left great toe extension strength of 4/5.

The patient was a 72-year-old man who was admitted with a 2-year history of low back pain accompanied by left lower extremity radicular pain and neurogenic claudication. He developed radiating pain involving the lateral border of the left thigh and the calf after walking 100 meters. The mean visual analog scale (VAS) score for low back pain was 7, and the mean VAS score for radiating pain in the lateral border of the left thigh and the calf was 6. The modified Japanese Orthopaedic Association (M-JOA) score was 28, and the Oswestry Disability Index (ODI) was 41. Physical examination revealed a positive lumbar hyperextension test, mild atrophy of the left thigh muscles, a positive left femoral nerve stretch test, diminished left patellar reflex, left extensor hallucis longus (EHL) muscle strength graded 4/5, and a negative left straight leg raise test.

## Diagnosis and surgical treatment

**Figure 1:**
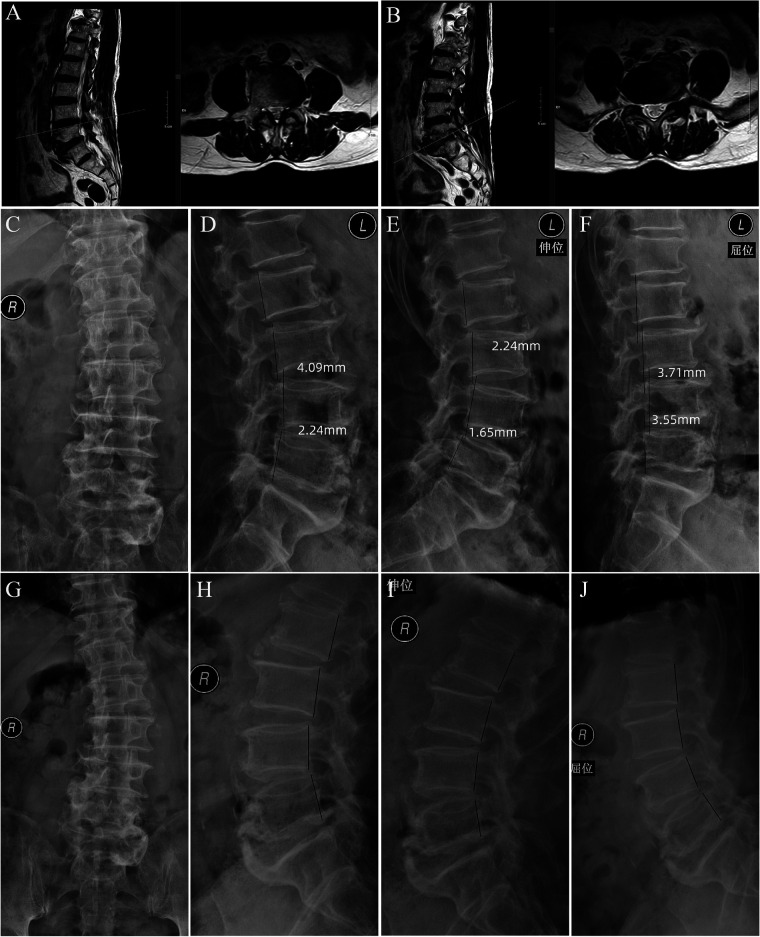
A 72-year-old male patient diagnosed with multilevel lumbar spinal stenosis and grade I spondylolisthesis based on clinical symptoms and imaging findings, treated with the contralateral trans-spinous base inclined (CTI)-AUSS approach. **(A)** Preoperative L3-4 sagittal and axial MRI images. **(B)** Preoperative L4-5 sagittal and axial MRI images. **(C–F)** Preoperative AP, lateral, flexion, and extension radiographs. **(G–J)** Postoperative AP, lateral, flexion, and extension radiographs. (AP, anteroposterior).

Clinical and radiographic evaluation by three senior spine surgeons and three radiologists revealed mild lumbar scoliosis, grade I anterolisthesis at L3 (Figures 1C–F), and MRI findings of left lateral recess stenosis at L3/4 and L4/5 with associated disc herniation, ligamentum flavum hypertrophy, facet arthropathy, and a far-lateral disc herniation at L4/5 compressing the exiting left L4 nerve root (Figures 1A,B).

The patient had no significant past medical history, no underlying comorbidities, and no cardiovascular or cerebrovascular diseases. After discussing the condition, the AUSS technique, and associated risks with the patient and his family, informed consent was obtained. The CTI-AUSS procedure was performed on October 15, 2025. Total operative time was 240 min, with an estimated blood loss of 68 mL by Hct calculation method (5). The patient was ambulatory by postoperative day 3 and discharged on day 5.

## Surgical technique

**Figure 2:**
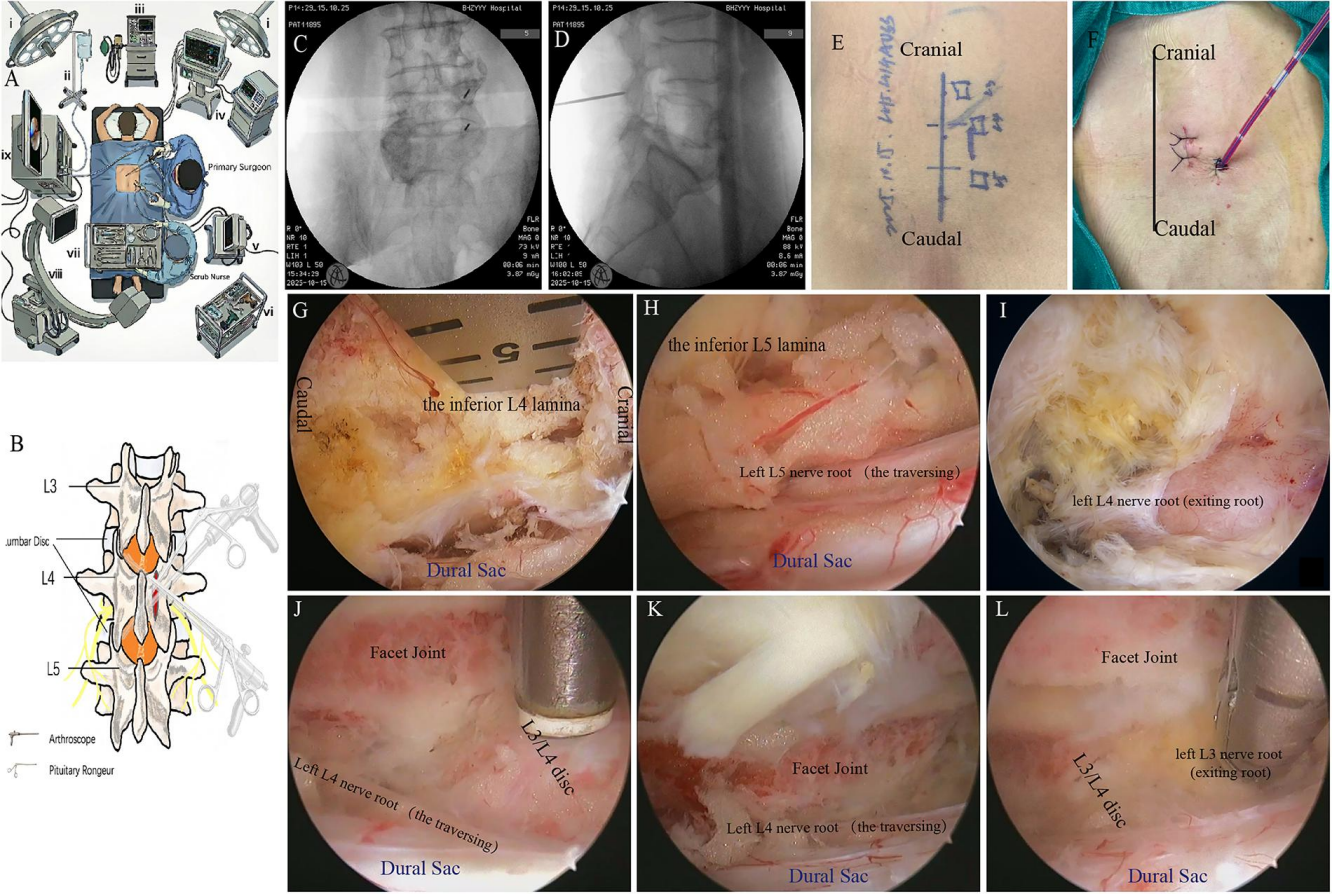
CTI-AUSS surgical schematic. **(A)** With the patient's symptoms localized to the left side, the surgeon stood on the right and operated on the left-sided pathology using an inclined surgical trajectory (right-handed surgeon posture). i: Surgical Light. ii: Irrigation Fluid. iii: Anesthesia Machine. iv: ECG/EKG. v: Radiofrequency (RF) Plasma Electrode. vi: Surgical Power System. vii: Instrument Cart. viii: C-arm Fluoroscopy System. ix: Arthroscopy System. **(B)** Schematic of the inclined surgical trajectory. (The designs of A and B originated from PowerPoint and Gemini (e.g., Google Gemini) **(C)** Intraoperative AP C-arm. **(D)** Intraoperative lateral C-arm. **(E)** Preoperative skin marking. **(F)** Postoperative incision with drain. **(G)** Osteotome removing L4 lamina. **(H)** Decompression of the left traversing L5 nerve root within the lumbar spinal canal was achieved. **(I)** Decompression of left L4 nerve root (exiting root) in the lateral recess was performed. **(J)** Compression of the left traversing L4 nerve root within the lumbar spinal canal at the L3/4 level was observed. **(K)** Decompression of the left L4 traversing nerve root in the lumbar spinal canal at the L3/4 level was performed. **(L)** Decompression of left L3 nerve root (exiting root) in the lateral recess was performed.

1. Positioning and Localization: With the patient's symptoms localized to the left side, the surgeon stood on the right (Figure 2A). A contralateral trans-spinous inclined trajectory was used to access the left-sided pathology (Figure 2B). C-arm was used in the anteroposterior(AP) view to identify the medial pedicle line on the right at L3, L4, and L5 (6) (Figures 2C,D). Transverse lines were marked at the L3/4 and L4/5 disc spaces. A 3 cm longitudinal incision was marked at the intersection of these lines with the medial pedicle line (Figure 2E). Standard preparation and draping were performed.
2. Incision and Channel Creation: A 3 cm skin incision was made, followed by sequential dilation and placement of the working channel.
3. Under endoscopic visualization, soft tissue was cleared using radiofrequency ablation at L4/5. After confirming the level with C-arm, a burr was used to thin the lamina from caudal to cranial and medial to lateral, starting at the junction of the spinous process base and the inferior L4 lamina. A Kerrison punch was used to resect the lamina up to the proximal ligamentum flavum insertion (Figure 2G). Drilling continued from the superior L5 lamina to the distal ligamentum flavum insertion. The ligamentum flavum was dissected from the dura using a nerve hook and resected, exposing the dural sac and the contralateral L5 nerve root. No significant disc herniation was noted. Contralateral decompression was carried to the medial pedicle wall. Partial resection of the spinous process base allowed access to the ipsilateral side, where hypertrophied ligamentum flavum was removed and the ventral aspect of the superior articular process was drilled, achieving decompression to the ipsilateral medial pedicle wall and exposing the ipsilateral L5 nerve root (Figure 2H). The tip of the superior articular process was partially drilled, and the ligamentum flavum was resected to expose and decompress the ipsilateral foramen and the lateral region of the L4 nerve root (Figure 2I). Complete decompression of the dural sac and bilateral nerve roots was confirmed. Absorbable gelatin hemostatic matrix was applied.
4. Through the same skin incision, the working channel was angled cephalad and laterally. Radiofrequency ablation was used to clear the soft tissue over the L3/4 interlaminar space. The level was reconfirmed with C-arm fluoroscopy. Starting from the junction of the spinous process base, the inferior lamina of L3, and the superior lamina of L4, a burr was used to thin the lamina from caudal to cranial and from medial to lateral. A Kerrison punch was then used to resect the lamina up to the proximal attachment of the ligamentum flavum. Drilling continued from the superior edge of the L4 lamina to the distal insertion of the ligamentum flavum. A nerve hook was used to dissect the ligamentum flavum from the dural sac, and the thickened ligamentum flavum was resected with a Kerrison punch, exposing the dural sac and the contralateral (healthy side) L4 nerve root. No significant disc herniation was observed. Contralateral decompression was carried to the medial wall of the pedicle. Partial bone resection at the base of the spinous process was performed, and the thickened ligamentum flavum on the ipsilateral (symptomatic side) was removed with a Kerrison punch. The ventral aspect of the hypertrophic superior articular process was drilled, achieving decompression to the ipsilateral pedicle medial wall and exposing the ipsilateral L4 nerve root (Figures 2J,K). The tip of the superior articular process was partially drilled, and the ligamentum flavum was resected to expose and decompress the ipsilateral neural foramen and the lateral region of the L3 nerve root (Figure 2L). Complete decompression of the dural sac and bilateral nerve roots was confirmed. Absorbable gelatin hemostatic matrix was applied to reduce postoperative bone surface oozing.
5. Closure: A drain was placed over the lamina, and the incision was closed in layers (Figure 2F).

## Outcomes

By postoperative day 3, the patient was ambulatory with minimal incisional discomfort and significant improvement in left leg radicular pain (VAS 2). At the 3-month follow-up, low back pain had completely resolved (VAS 0), with no claudication or radicular symptoms (VAS 1). Left thigh atrophy persisted, but the femoral nerve stretch test was negative, the patellar reflex was normal, and great toe extension strength was 5/5. The M-JOA score improved to 5 (an 82.14% improvement rate), and the ODI was 4, reflecting near-complete resolution of symptoms, normal lumbar function, and a straight leg raise of 70°.

Preoperative dynamic x-rays showed 3.55 mm of anterolisthesis at L3 (Meyerding Grade I) (Figures 1C–F). At 3 months postoperatively, follow-up x-rays demonstrated no progression of the listhesis. A slight increase in lumbar curvature was noted on the AP view (Figures 1G–J), possibly related to postural changes after symptom resolution.

**Figure 3:**
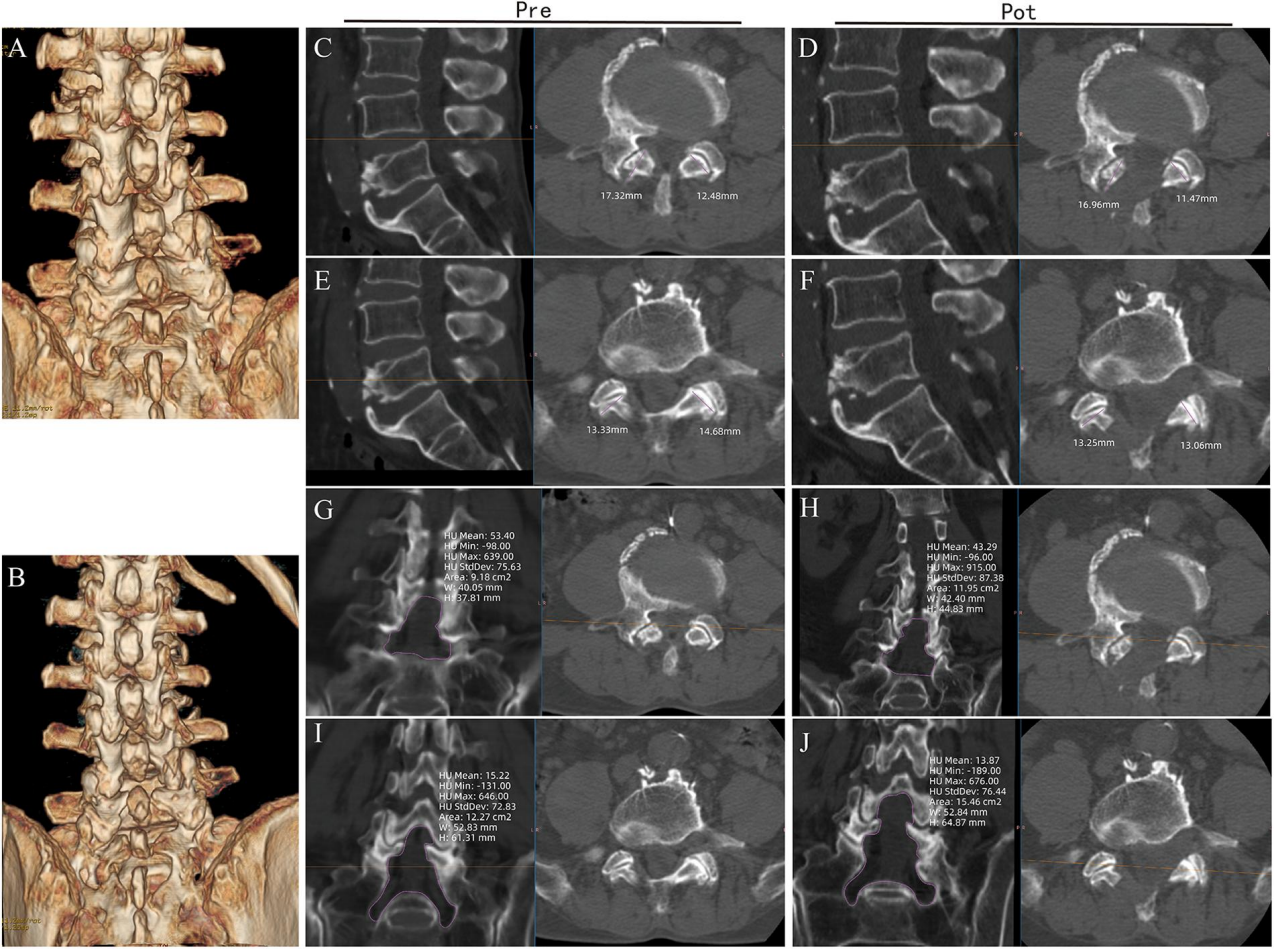
Preoperative vs. postoperative imaging. **(A,B)** 3D CT reconstruction: preop (A) and postope (B). **(C,D)** L3-4 sagittal and axial views: preop (C) and postop (D). **(E,F)** L4-5 sagittal and axial views: preop (E) and postop (F). **(G,H)** L3-4 coronal and axial views: preop (G) and postop (H). **(I,J)** L4-5 coronal and axial views: preop (I) and postop (J). Preop:preoperative. Postop:postoperative.

Postoperative MRI and CT confirmed adequate decompression of the left lateral recess and neural foramen at both levels. Preservation rates of facet joint calculated on CT as (postoperative length/preoperative length) × 100%, were 95.61% and 91.91% at L3-4 (Figures 3C,D), and 99.40% and 88.96% at L4-5 (Figures 3E,F). The laminectomy area (7) on 3D CT reconstruction was 2.77 cm^2^ at L3-4 (Figures 3G,H) and 3.19 cm^2^ at L4-5 (Figures 3I,J).

**Figure 4:**
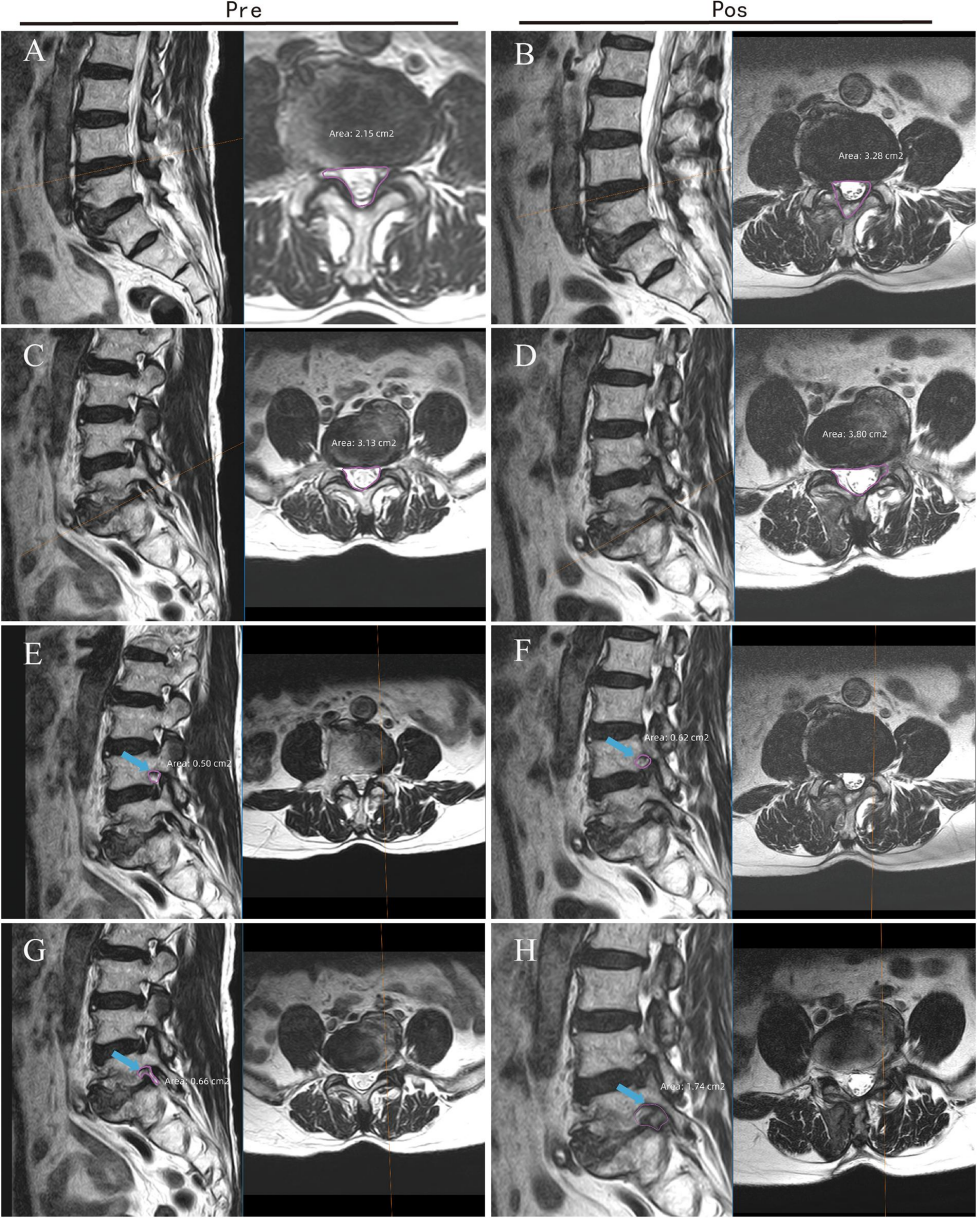
Preoperative vs. postoperative MRI. **(A,B)** L3-4 sagittal and axial canal areas: preop (A) and postop (B). **(C,D)** L4-5 sagittal and axial canal areas: preop (C) and postop (D). **(E,F)** L3-4 sagittal foraminal areas and axial views: preop (E) and postop (F). **(G,H)** L4-5 sagittal foraminal areas and axial views: preop (G) and postop (H).

Expansion rates of spinal canal(ER-SC) = (postoperative spinal canal area - preoperative spinal canal area)/preoperative spinal canal area  × 100% (8) on MRI were 52.56% at L3-4 (Figures 4A,B) and 21.41% at L4-5 (Figures 4C,D). Expansion rates of sagittal foramen area were 24.00% at L3-4 (Figures 4E,F) and 160.01% at L4-5 (Figures 4G,H). All measurements were independently performed by three spine surgeons using PACS, with the averages used for analysis. After partial resection of the inferior lamina for decompression, the posterior bony wall of the spinal canal overlying the dura was removed, resulting in spinal canal enlargement, and marked enlargement of the spinal canal at the L3/4 level was clearly observed. The patient's clinical symptoms were mainly due to severe compression at left L4 nerve root (exiting root), without evident clinical involvement of the L3 nerve root. Accordingly, the L4 nerve root was adequately decompressed during surgery, whereas the left L3/4 intervertebral foramen was not fully decompressed. Postoperatively, significant enlargement of the L4/5 intervertebral foramen was noted.

## Discussion

### Challenges in treating multilevel LSS in the elderly

LSS is a common degenerative condition involving disc herniation, ligamentum flavum hypertrophy, and facet arthropathy. These changes lead to spinal canal narrowing and compression of neurovascular structures, causing back pain, leg pain, neurogenic claudication, and muscle atrophy (9). Traditional laminectomy, while effective, carries inherent risks of infection and iatrogenic instability (10). Although posterior lumbar interbody fusion can address instability, it may accelerate adjacent segment degeneration (11).

In recent years, minimally invasive UBE techniques have been widely adopted for lumbar degenerative diseases. However, UBE's dual-channel design limits instrument maneuverability and visualization (12–14). Arthroscopic-assisted uni-portal spinal surgery (AUSS), introduced by En Song (6) in 2021, combines the wide visualization of UBE, the minimally invasive nature of coaxial endoscopy, and the triangulation principles of arthroscopy. This technique enables dual-channel access through a single 8–10 mm incision using a 30°arthroscope. AUSS offers improved rotational flexibility compared to UBE, with reduced total incision length and intraoperative blood loss. Its integration of arthroscopic instruments facilitates advanced procedures such as percutaneous lumbar interbody fusion (15), ligamentum flavum suspension, dural repair, and annulus fibrosus suture with bone anchor fixation (16).

Given its relatively recent introduction, long-term clinical outcomes for multilevel stenosis treated with AUSS require further investigation. Additionally, most multilevel applications have involved decompression without fusion—a potential concern given the frequent association of multilevel stenosis with instability.

### Challenges of surgical approaches to lateral recess and foraminal stenosis

The combination of disc herniation, facet osteophytes, ligamentum flavum buckling and hypertrophy, and spondylolisthesis leads to narrowing of the central canal, neural foramen, and lateral recess (9). Multilevel stenosis often involves combined pathology at multiple sites.Although percutaneous transforaminal endoscopic discectomy (PTED) is minimally invasive, its decompression efficiency is limited in cases of extensive bony hypertrophy. Li et al. (17) compared AUSS with PTED for far-lateral lumbar disc herniation and found that AUSS was superior in pain relief, functional improvement, and complication reduction, suggesting its unique advantages in complex lumbar degenerative diseases.

The interlaminar approach is the most common UBE approach for LSS. Xu et al. (4) reported the S-UBE technique for adjacent two-level LSS, with 95.12% excellent or good outcomes and a mean facet preservation rate of 84.76%. However, lateral recess decompression required more extensive bone resection. Wang et al. (6) retrospectively found that both AUSS and UBE achieved good clinical outcomes for LSS, but AUSS offered better maneuverability. Nevertheless, AUSS based on the ipsilateral approach still requires substantial facet joint resection for adequate decompression, leading to iatrogenic instability.

Bayram et al. (18) reported full-endoscopic unilateral laminotomy for bilateral decompression (LE-ULBD) in patients with grade I degenerative spondylolisthesis: 90.6% achieved good outcomes, but 28.1% showed progression of listhesis, and one patient required fusion after two-level decompression. Multilevel decompression without fusion carries inherent instability risks (19), necessitating long-term follow-up.

The ipsilateral UBE or AUSS approach struggles to simultaneously decompress the lateral recess and foramen without extensive facet resection, which can destabilize the spine. The contralateral approach preserves midline structures and interspinous ligaments. In recent years, the contralateral inclinatory approach has been used for bilateral decompression. Tian et al. (20) described the contralateral inclinatory approach using UBE (CIA-UBE) for lateral recess and same-level foraminal lesions. However, CIA-UBE requires two separate incisions on the contralateral side, increasing paraspinal muscle dissection and causing instrument collision in the deep narrow space. It is mainly suitable for single-level, simple unilateral lateral recess stenosis and has limited efficacy for central stenosis, spondylolisthesis, or far-lateral disc herniation.

To overcome these limitations, our team designed and applied the contralateral trans-spinous base inclined approach using AUSS (CTI-AUSS). It is important to clarify that “trans-spinous” does not mean complete resection of the spinous process, but rather partial resection of the spinous process base. This minor bony modification creates a safe contralateral inclined working channel without compromising the biomechanical integrity of the spinous process and interspinous ligament, thus avoiding postoperative iatrogenic instability.

### CTI-AUSS advantages and limitations

Technically, CTI-AUSS differs essentially from CIA-UBE: CTI-AUSS uses a single incision crossing the spinous midline, with instruments entering through the junction of the spinous process base and the adjacent lamina. It first achieves contralateral (healthy side) ligamentum flavum and lamina resection, then partially resects the ipsilateral (symptomatic side) spinous process base to reach the ipsilateral lateral recess, enabling single-incision, same-level bilateral lateral recess decompression and spinal canal enlargement. Because AUSS uses a single-port coaxial endoscope with a 30° arthroscope, two instruments can operate within a single flexible channel, avoiding the instrument interference seen in dual-channel techniques and allowing greater maneuverability.

In terms of indications, CTI-AUSS has a broader range than CIA-UBE. Recent evidence suggests that in patients with grade I spondylolisthesis or one- to two-level stenosis, long-term outcomes of decompression alone are comparable to those of decompression with fusion (21, 22). Therefore, simple decompression was appropriate for this patient. Using CTI-AUSS, we successfully achieved adequate two-level decompression (L3/4 and L4/5 left lateral recess and foraminal stenosis involving L3, L4, and L5 nerve roots). Postoperative imaging confirmed a mean facet joint preservation rate of 93.97% (L3-4: 95.61% and 91.91%; L4-5: 99.40% and 88.96%). This “asymmetric decompression” strategy completely relieved nerve compression while providing excellent biomechanical stability. Finite element analysis by Li et al. (23) also supports that, under equivalent decompression, the contralateral approach offers superior spinal biomechanical stability compared with the ipsilateral approach. Three-month follow-up showed no progression of listhesis and a slight improvement in lumbar curvature. These results suggest that for elderly patients with multilevel lateral recess and foraminal stenosis, especially those with grade I spondylolisthesis or severe facet hypertrophy, CTI-AUSS achieves adequate neural decompression while maximally preserving posterior stabilizing structures, reducing the risk of postoperative iatrogenic instability and listhesis progression.

In summary, CTI-AUSS inherits the advantages of single-port coaxial endoscopy and, through partial resection of the spinous process base, establishes a safe and effective contralateral inclined working pathway. Compared with CIA-UBE, CTI-AUSS offers broader indications, less muscle dissection, better instrument maneuverability, and higher facet joint preservation rates, making it particularly suitable for elderly patients with mild spondylolisthesis or multilevel degeneration.

Nevertheless, this report has limitations. First, it is a single case report; its clinical representativeness and technical generalizability require validation by large cohort studies. Second, although the CTI trajectory effectively protects the ipsilateral facet joint, the potential long-term impact of contralateral partial bone resection at the spinous process base and lamina on spinal stability remains unclear. Finally, the short 3-month follow-up is insufficient to fully assess the risk of adjacent segment degeneration or recurrent listhesis. Future prospective, multicenter, long-term studies with dynamic radiography and three-dimensional gait analysis are needed to evaluate the long-term efficacy of CTI-AUSS in complex multilevel LSS.

## Conclusion

This study describes the first application of the CTI-AUSS technique for treating multilevel lumbar lateral recess and foraminal stenosis in an elderly patient. By comparing clinical symptoms, radiographic findings, and spinal stability outcomes, we found that the CTI-AUSS technique is a safe and effective surgical option. It provides excellent clinical and radiographic results while preserving spinal stability, protecting paraspinal muscles and facet joints, and reducing the risk of postoperative spondylolisthesis or instability.

## Data Availability

The original contributions presented in the study are included in the article/Supplementary Material, further inquiries can be directed to the corresponding authors.
